# Supplementary material for: Prolonged or Transition to Metabolically Unhealthy Status, Regardless of Obesity Status, Is Associated with Higher Risk of Cardiovascular Disease Incidence and Mortality in Koreans
Source: Nutrients. 2022 Apr 14;14(8):1644. doi: 10.3390/nu14081644 (PMC9028697; doi:10.3390/nu14081644)
Supplement: Supplementary file 1 [file nutrients-14-01644-s001.zip › nutrients-1667449-supplementary.pdf]

Table S1. Hazard ratios (HRs) and 95% confidence intervals (CIs) for cardiovascular, cancer, and all-cause mortality according to the number of metabolic health risks and obesity status in KNHANES participants**.**

Number of metabolic health risks (Weighted N)	Cardiovascular mortality	Cancer mortality	All-cause mortality
**All participants**			
0 (n=9,038,503)	1.00 (ref)	1.00 (ref)	1.00 (ref)
1 (n=6,302,879)	1.67 (1.25-2.22)	0.99 (0.79-1.24)	1.11 (0.97-1.27)
2 (n=2,536,693)	1.61 (1.12-2.32)	1.04 (0.79-1.37)	1.23 (1.04-1.46)
3 (n=532,714)	2.58 (1.53-4.36)	1.25 (0.77-1.92)	1.61 (1.24-2.08)
P for trend	<0.001	0.530	<0.001
**Non-obese**			
0 (n=6,703,404)	1.00 (ref)	1.00 (ref)	1.00 (ref)
1 (n=3,683,304)	1.91 (1.36-2.68)	0.85 (0.65-1.10)	1.13 (0.97-1.32)
2 (n=1,223,957)	1.47 (0.90-2.39)	1.08 (0.78-1.49)	1.36 (1.11-1.68)
3 (n=226,710)	2.45 (1.19-5.04)	1.49 (0.83-2.67)	1.82 (1.31-2.53)
P for trend	0.006	0.514	<0.001
**Obese**			
0 (n=2,335,099)	1.00 (ref)	1.00 (ref)	1.00 (ref)
1 (n=2,619,575)	1.35 (0.68-2.70)	1.76 (1.09-2.85)	1.29 (0.94-1.77)
2 (n=1,312,736)	1.95 (0.98-3.90)	1.38 (0.80-2.40)	1.29 (0.93-1.80)
3 (n=306,004)	2.96 (1.12-7.22)	1.30 (0.60-2.83)	1.71 (1.10-2.67)
P for trend	0.007	0.532	0.031

Non-obese and obese were categorized as body mass index < 25 kg/m^2 and ≥ 25 kg/m^2, respectively. Values are presented as the HRs and 95% CI. The HRs (95% CI) were calculated using a Cox proportional hazard regression model adjusting for age, sex, residence area, education level, household income, smoking status, drinking status, metabolic equivalent of task.

Table S2. Hazard ratios (HRs) and 95% confidence intervals (CIs) for the all-cause mortality and cause-specific mortality according to the combination of metabolic health risks and obesity status by sex in KNHANES

	Men			Women		
	Weighted event (N)	Weighted follow-up (PY)	HR (95% CI)	Weighted event (N)	Weighted follow-up (PY)	HR (95% CI)
**Cardiovascular mortality**						
MHN	21,479	23,745,670	1.00 (ref)	13,171	30,134,350	1.00 (ref)
MUN	58,636	19,908,434	1.99 (1.28-3.11)	43,965	19,967,306	1.48 (0.93-2.35)
MHO	4,273	9,687,018	0.95 (0.39-2.36)	2,229	9,310,493	0.60 (0.28-1.30)
MUO	15,938	17,734,072	1.07 (0.66-1.75)	30,031	16,001,223	1.64 (1.03-2.62)
**Cancer mortality**						
MHN	60,141	23,745,670	1.00 (ref)	34,248	30,134,350	1.00 (ref)
MUN	78,816	19,908,434	1.05 (0.78-1.42)	40,798	19,967,306	0.76 (0.50-1.17)
MHO	7,409	9,687,018	0.56 (0.31-1.00)	6,310	9,310,493	0.54 (0.28-1.03)
MUO	40,928	17,734,072	1.05 (0.74-1.49)	36,481	16,001,223	0.90 (0.59-1.37)
**All-cause mortality**						
MHN	163,293	23,745,670	1.00 (ref)	78,644	30,134,350	1.00 (ref)
MUN	258,999	19,908,434	1.26 (1.05-1.52)	168,964	19,967,306	1.09 (0.86-1.38)
MHO	25,727	9,687,018	0.70 (0.48-1.03)	14,945	9,310,493	0.59 (0.39-0.89)
MUO	91,269	17,734,072	0.84 (0.68-1.04)	111,107	16,001,223	1.00 (0.78-1.29)

Values are presented as the HRs and 95% CI. The HRs (95% CI) were calculated using a Cox proportional hazard regression model adjusting for age, sex, residence area, education level, household income, smoking status, drinking status, metabolic equivalent of task. BMI: body mass index; KNHANES: Korea National Health and Nutrition Examination Survey; MH: metabolic health; MHN: metabolically healthy non-obese (BMI <25 kg/m^2 & 0 metabolic health risk); MHO: metabolically healthy and obese (BMI ≥25 kg/m^2 & 0 metabolic health risk); MUN: metabolically unhealthy non-obese (BMI <25 kg/m^2 & 1-3 metabolic health risks); MUO: metabolically unhealthy obese (BMI ≥25 kg/m^2 & 1-3 metabolic health risks); PY: person-years.
